# A Fatal Tyre Blast Injury: An Autopsy Case

**DOI:** 10.3126/nje.v12i1.41186

**Published:** 2022-03-31

**Authors:** Rajesh Kumar, Nishat Ahmed Sheikh

**Affiliations:** 1 Department of Forensic Medicine and Toxicology, All India Institute of Medical Sciences, Deoghar, India; 2 Department of Forensic Medicine and Toxicology, All India Institute of Medical Sciences, Gorakhpur, India

**Keywords:** Autopsy, Blast Injury, Tyre Blast Injuries, Tyres, Multiple trauma

## Abstract

A 49-year-old man sustained an accidental injury when he was changing and inflating the tyre of a truck, and there was a sudden explosion of the truck tyre at the service station, which was by the roadside of the highway. With the pressure of air generated due to the exploding tyre, the victim was blown to around 6 feet away. He was declared dead on admission. The medico-legal examination was conducted, and death was determined to be multiple organ injuries [mainly head, chest, and abdominal injuries] caused by the shock wave produced due to tyre explosion. Tyre blast injuries are not so common. A meticulous post-mortem examination is fundamental in formulating and recording the pattern of traumatic injuries. Preventive occupational measures should be put in place.

## Background

Tyre burst injuries are relatively rare but are known for causing severe injury patterns. Tyres can be thought of as air tanks, but the same safety rules that apply to pressure tanks do not apply to tyres. Blast overpressure is described as an increase in pressure above atmospheric values, as witnessed in a blast of explosive weapons. Air compression before the blast wave accelerates air molecules and generates a pressure peak [1-3]. This high pressure lasts milliseconds before dropping to sub-atmospheric levels and returning to ambient pressure and is referred to as a shock wave [4, 5].

The tyre explosion has a high potential for destruction, yet it has garnered little attention in the medical literature, [6] commonly such tyre burst occurs while servicing on the highway or in service stations [7]. The severity of the injury is determined by the size of the tyre, air pressure, and the distance between the victim and the tyre [8]. Usually, mechanics and bystanders get affected and are victims by a burst of tyres. A burst of tyres may result in high morbidity and mortality, however, there is not much data on deaths resulting from the blast of tyres [9]. Injuries due to burst tyres are direct injuries due to metal rim fragments along with barotraumas due to high pressures resulting in body surface displacements; an internal pressure wave is also generated due to acceleration of body surface leading to differential pressure in body tissue and viscera [10]. Ear, gut, and lungs are the commonly affected organs due to the primary blast injury. When fragments of the tyre or other parts of the wheel strike the body due to acceleration generated by initial detonation or overpressure loads, such injuries are referred to as secondary blast injuries [11, 12]. The blast wave causes displacement of the body or parts of the body, which can result in avulsion on impact with solid objects, resulting in tertiary blast injuries. Literature has shown maxillofacial, cranial injuries, and fractures of the long bones caused by rim pieces. Similarly, tympanic perforation, eye injury, and more severe injuries such as oesophageal rupture, pneumomediastinum can be caused due by barotraumas [13]. A few cases had been reported on this issue [14]. This study describes the unintentional death of a middle-aged mechanic caused by a tyre blast at the workplace. The incidence site and gross findings associated with tyre blast wave injuries are discussed.

### Case Presentation

A 49-years-old man sustained an accidental injury on the 31st of December when he was changing and inflating the tyre of a truck and there was a sudden explosion of the truck tyre at the service station which was by the roadside of the highway. With the pressure of air generated due to the exploding tyre, the victim was blown to around 6 feet away. He was taken to the hospital in an unconscious state and was declared brought dead on admission at the All-India Institute of Medical Sciences in Delhi in the emergency department. The police were intimated and accordingly, the case was registered as a medicolegal case. Inquest papers were prepared by the Investigating Officer and the body was shifted to the Morgue associate with the Forensic Medicine and Toxicology Department at AIIMS, Delhi for medico-legal post-mortem examination.

### Autopsy Findings

The Incident occur at about 4:00 PM on 31st Dec. Postmortem conducted at 12:30 PM and concluded at 1:45 PM on 1st January. A detailed complete and meticulous post-mortem examination was performed, rigor mortis all over the body in the supine position, cadaveric lividity on the back, dependant parts of the body. The lividity was fixed. There was the presence of clotted blood and oozing on left ear ossicles (Fig. No 1a) in external meatus along with rupture of the tympanic membrane (Fig. No 1b) There was reddish contusion with overlying abrasion of size 2x1.5 cm and 5 x 2.5 cm over the left posterior aspect of the wrist and elbow joints, 4 x 2.2 cm over the dorsolateral aspect of the right wrist joint, and multiple reddish contusion with overlying abrasion of size varying from 0.5 x 0.2 cm to 5 x 1 cm over the anterior aspect of the chest involving an area of around 30 x 9 cm. The reddish contusion with overlying abrasion on the wrist and the elbow were caused by the rough surface and according to the eyewitnesses, the man was thrown around six feet away from the blasted tyre.

On internal examination, subarachnoid haemorrhage with underlying brain parenchyma, contusion in patches were present on both cerebral hemisphere (Fig. No 2), the chest cavity was filled with about 1000 mL fluid blood and about 500 gms clotted blood on both sides of the chest (Fig. No 3, lungs were collapsed partially and showed contusion. The right lung showed rupture and contusion at the hilum region and the weight of the lung was 265 gms. The left lung on the anterior surface and hilum region had a rupture along with contusion with eight of 273 Gm (Fig. No 4a). Contusion was appreciated at the base of the heart and the origin of the aorta (Fig. No 4b). The anterior surface of the liver was contused and rupture of the right lobe was seen (Fig. No 5a). The peritoneal cavity contained around 500 mL of fluid blood along with fecal matter. About 6 cm tear in the transverse colon with the expulsion of fecal matter (Fig. No 5b). In this case, the toxicological analysis of the viscera and blood did not reveal alcohol or any drug. Death, in this case, was due to multiple organ injuries [mainly head, chest, and abdominal Injuries] caused by the shock wave produced due to tyre explosion.

## Discussion

### Tyre Blast Injuries

The situations surrounding the incident, as well as the location of injuries anatomically, suggested that a middle-aged mechanic was injured accidentally as a result of a tyre explosion while changing and inflating at a service station along the highway. Because such work-related unintentional injuries are underreported, the forensic literature may be deficient in this regard. Multiple organ injuries [mainly head, chest, and abdominal injuries] caused by the shock wave produced due to tyre explosion was concluded as the cause of death. An inflated tyre contains a high amount of potential energy.

### Pattern of Injuries

A 63000 ft-lb of energy is released when a truck tyre inflated to 90 psi explodes. The estimated force is around 2000 g, which is enough to raise a 3000 lb car 21 ft off the ground [15]. The blast wave produced by the tyre explosion struck the thorax, causing lung injuries (lung showed rupture and contusion). Contusion at the base of the heart and the origin of the aorta. These observations dubbed the primary consequence of the blast wave, Furthermore, primary blast overpressure was linked to abdominal visceral injuries (contusion and rupture of the right lobe of the liver, rupture of the transverse colon). The movement of the body on the ground as well as environmental structures due to explosion caused Subarachnoid haemorrhage. In fatal cases, 47 % of injuries were caused by a primary blast, with the head and thorax being particularly affected. Brain injuries can be direct or indirect, such as a cerebral contusion, Subdural and subarachnoid haemorrhage are common in fatal cases [16]. Even if no other visible evidence of damage to the thoracic region is present, a fatal primary blast lung injury can occur [17]. Secondary injuries are caused by flying items, pieces, and materials driven by the blast, such as the rim and ring. The body collapsing and striking the ground or other close objects causes tertiary injuries [18, 19].

In literature there have been instances in certain cases wherein a tyre burst did not result in a fatality, however, reports of cases with fatal incidents have been reported by certain authors. Fatal pneumatically induced injuries are rare in forensic literature; high-energy released due to blast of tyre results in fatal injuries [20].

Deforming injuries and fatalities caused by explosions among mechanics servicing trucks, in the existing literature, it’s only been mentioned a few times. The size of the tyre’s air pressure and the distance between the tyre and the victim at the moment of the blast influence the severity of the injury. Explosions are common during tyre servicing, particularly when inflating the tyres; bystanders are also injured and may sustain fatal injuries [21, 22].

## Conclusion

A tyre burst can happen while servicing or inflating tyres. High inflating pressure and a short safety distance are the two most common causes of fatal injuries. The high energy produced by a large tyre burst can result in severe injuries with high morbidity and mortality. A meticulous post-mortem examination is fundamental in formulating and recording the pattern of traumatic injuries. Preventive occupational measures should be put in place.

## Figures and Tables

**Figure 1 (a & b):**
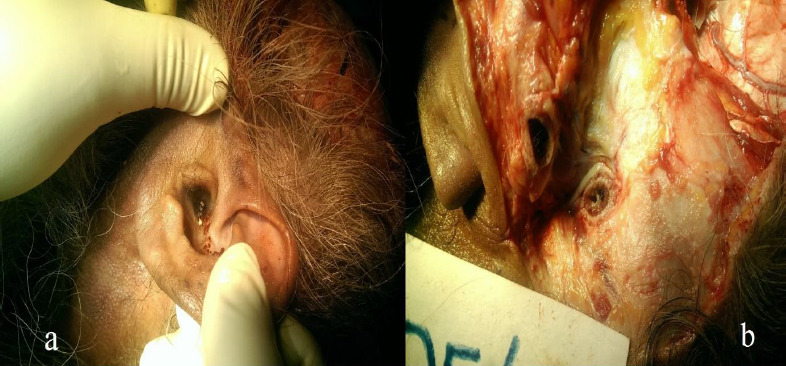
a. Blood clot with ear ossicles present in external meatus of left ear. b. Rupture of tympanic membrane.

**Figure 2:**
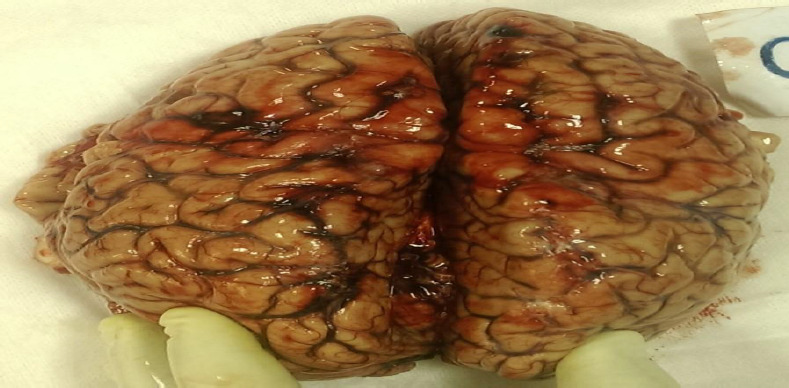
Brain: Presence of subarachnoid haemorrhage

**Figure 3:**
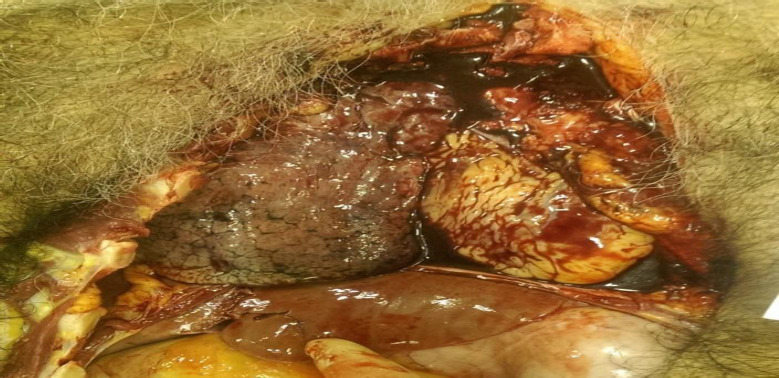
Thoracic cavity filled with blood

**Figure 4 (a & b):**
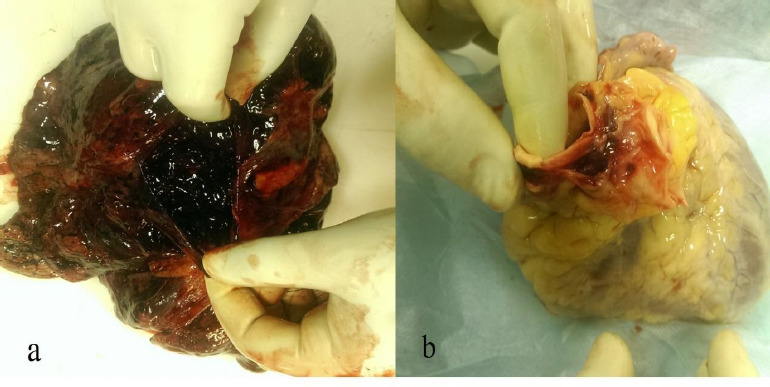
a. left lung: Contusion with rupture present. b. Heart: Contusion at origin of aorta.

**Figure 5 (a & b):**
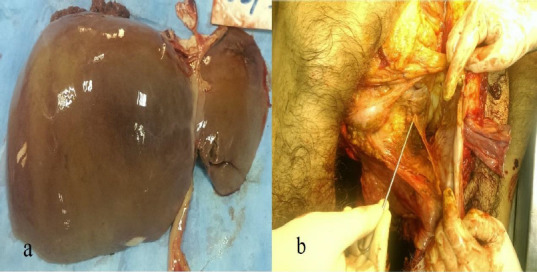
a. Liver: Contusion over anterior surface of right lobe and rupture of left lobe. b. Rupture of intestinal loop
